# Meningitis and Lumbar Puncture

**Published:** 1903-09-26

**Authors:** 


### MENINGITIS AND LUMBAR PUNCTURE.

The chief points which would lead one to the diagnosis of meningitis in any patient are headache, drowsiness with irritable and noisy intervals, vomiting, retraction of the head, squint, rigidity of limbs and presence of Kernig's sign, a condition in which it is impossible to completely extend the leg on the thigh when the latter is flexed to make a right angle with the trunk. The symptoms usually come on rather rapidly, and may be accompanied by high fever and a very frequent irregular pulse. The anterior fontanelle when open is usually bulging. These signs are the ordinary concomitants of almost all forms of meningitis. In the post-basic variety the retraction of the head is usually more marked.

When the exudation is purulent—as in pneumococcus cases—there is little retraction of the head and the symptoms may be quite obscure until the last few hours of life. The only satisfactory method of making a differential diagnosis between the various forms of meningitis is by an examination of the subarachnoid fluid, and even this does not always yield conclusive results.[1] To obtain this fluid a quite simple and harmless operative procedure is necessary. In the majority of cases of cerebral and cerebro-spinal meningitis the subarachnoid space is filled with fluid under considerable pressure which can be drawn through a hollow needle inserted in the lumbar region. The skin over the last dorsal and first two or three lumbar vertebræ is cleaned with soap and water and ether, followed by some antiseptic lotion such as 1—40 carbolic, and then a drop of pure carbolic acid is applied to the skin over the space between the first and second lumbar spines. The puncture should be made with the needle of a glass hypodermic syringe. In the case of any but very young infants a needle two or more inches in length is necessary. The syringe and needle are boiled immediately before use, and the needle is inserted just to one side of the middle line, in the spot rendered white by the carbolic acid application, and directed slightly upwards and inwards, *i.e.*, towards the middle line passing between the vertebral laminæ to the spinal membranes until the fluid wells up into the syringe, when it is a good plan to disconnect the barrel and allow the fluid to drip from the needle into a boiled test-tube. If the fluid is clear and remains so on standing it is probably normal cerebro-spinal fluid, containing very little albumen, reducing Fehling's solution and without appreciable sediment. Should the fluid be clear but form a coagulum on standing it is usually obtained from a case of meningeal inflammation; it then contains albumen in fair quantity, does not reduce Fehling's solution, and has a deposit which when suitably stained and examined microscopically may show tubercle bacilli, the diplococcus meningitidis of post-basal meningitis (an intracellular organism easily stained by methylene blue), or some other form of bacterium, the finding of which may settle the diagnosis so far as the meningitis is concerned. Occasionally the cerebro spinal fluid is turbid, full of pus cells, and swarming with pyogenic organisms. Chief among these are the pneumococcus and varieties of staphylococci. Finally, if clear fluid is drawn off, which, on standing shows a diaphanous coagulum, on boiling shows excess of albumen and from which bacteria cannot be cultivated, it is strong presumptive evidence that the case is one of tuberculous meningitis.

The treatment of meningitis is so unsatisfactory that many practitioners consider a case hopeless when they have diagnosed an inflammation of the meninges from almost any of the various and numerous causes. That the prognosis is very grave cannot be denied, but it is by no means true that every case of meningitis is hopeless. Cases of the tuberculous form rarely recover, and those which have recovered are either mentally unsound, have some of the special senses impaired or are very liable to recurrence of the malady. In every instance of meningeal inflammation the patient should be kept very quiet in a darkened room. The head may be shaved, and kept cool with iced Leiter's tubes or an ice bag, and in the case of a child with dilated postauricular veins leeching over these is useful. The ears should be examined and, if bulging, the tympanic membrane must be punctured. In cases where otorrhœa is present this must be vigorously treated. Specific drug treatment is not satisfactory, but mercury seems to have been the drug most often used in those cases which have got well. The diet must be light, but as nourishing as possible, and when the patient is wasting rapidly cod liver oil or olive oil should be rubbed into the skin. To reduce the excessive intra-cranial pressure which ultimately kills in the large majority of cases, by causing failure of respiration, paracentesis of the spinal canal should be practised and repeated frequently. This prolongs the patient's life and gives his tissues a better chance to overcome the invading disease germs. A child who is quite unconscious and unable to take any nourishment may be thus brought back to a condition of semi-consciousness, and even when he has ceased to breathe the respiratory centre sometimes resumes its functions if lumbar puncture is immediately performed and the pressure relieved.[2]

[1] Brit. Med. Jour., May 2, 1903. [2] Ibid., July 11, 1903.

